# Disgust as an adaptive system for disease avoidance behaviour

**DOI:** 10.1098/rstb.2010.0117

**Published:** 2011-02-12

**Authors:** Valerie Curtis, Mícheál de Barra, Robert Aunger

**Affiliations:** The Hygiene Centre, London School of Hygiene and Tropical Medicine, London WC1E 7HT, UK

**Keywords:** infectious disease, disgust, evolutionary psychology, adaptive variation, hygiene behaviour, manners

## Abstract

Disgust is an evolved psychological system for protecting organisms from infection through disease avoidant behaviour. This ‘behavioural immune system’, present in a diverse array of species, exhibits universal features that orchestrate hygienic behaviour in response to cues of risk of contact with pathogens. However, disgust is also a dynamic adaptive system. Individuals show variation in pathogen avoidance associated with psychological traits like having a neurotic personality, as well as a consequence of being in certain physiological states such as pregnancy or infancy. Three specialized learning mechanisms modify the disgust response: the Garcia effect, evaluative conditioning and the law of contagion. Hygiene behaviour is influenced at the group level through social learning heuristics such as ‘copy the frequent’. Finally, group hygiene is extended symbolically to cultural rules about purity and pollution, which create social separations and are enforced as manners. Cooperative hygiene endeavours such as sanitation also reduce pathogen prevalence. Our model allows us to integrate perspectives from psychology, ecology and cultural evolution with those of epidemiology and anthropology. Understanding the nature of disease avoidance psychology at all levels of human organization can inform the design of programmes to improve public health.

## 1. Introduction: the problem of parasites

Parasites are ubiquitous; in some ecosystems their biomass rivals that of predators [1]. Parasitic viruses, bacteria, protozoa, nematodes, helminthes and arthropods live in durable relationships with their hosts, from whom they draw energy, shelter, transport and reproductive opportunity. They damage their host's inclusive fitness by producing toxins, manipulating behaviour to their own ends, and spreading to kin and community [2]. The costs of infection constitute an important selection pressure, which all animals face. As a result, natural selection has designed elegant and interlocking solutions to protect animals from parasite damage, including a range of physiological barriers and a complex immune system [3]. Beyond these physiological defences, animals also defend themselves from infection through behaviour that functions as a ‘behavioural immune system’ [4].

Pathogen avoidance behaviour has a long evolutionary history, and can be found in a broad range of taxa. For example, eusocial insects manage faecal wastes [5], crustaceans avoid diseased conspecifics [6], herbivores forage selectively to avoid faeces [7,8] and grooming behaviour is found in a range of species [9]. The disgust system is a psychological mechanism for producing pathogen avoidant behaviour [10–12]. In previous work, we have stressed the universality of disgust, showing that there is much that is similar about disgust responses between animals and humans, between humans and over historical time [11,13]. However, there is also much that differs between individuals and between social groups. In this paper, we look at disgust and disease avoidance behaviour in human individuals and in human social groups as an adaptive system. Natural selection has produced a solution to the problem of hard-to-detect parasites by designing a system that is sensitive to local information about infection risk. This system responds to parasite pressure not just over evolutionary time, but over lifetimes, using what cues it can. This may be information about an individual's current state, its history of sickness and exposure to disgusting experiences, or what it has learnt from the local culture and from the hygiene practices of others.

In this paper, we begin by recapping evidence that disgust is a universal driver of pathogen avoidance behaviour in humans. We then turn to sources of variation in the disgust system at the individual and group level. We set out the links between the hygiene behaviour of individuals and of groups and between individual disgust and the content of cultures. This allows us to integrate perspectives from psychology, ecology and cultural evolution, as well as epidemiological perspectives on disease prevalence, anthropological perspectives on manners and morality, and the symbolism of purity and pollution. Our conclusions stress the importance of using an evolutionary perspective in combination with interdisciplinary sources to provide an integrated understanding of the set of human behaviours that have a foundation in pathogen avoidance. This, may, in turn, offer insights that are important to the practice of public health.

## 2. The universality of disgust

Disgust is a fundamental part of human nature. Darwin was the first to propose that disgust is expressed universally [14] and many studies since then have supported this proposal [15,16]. Though there has been no systematic cross-cultural survey of the objects and events that elicit disgust in humans, the available data suggest that there is a universal set of disgust cues. These include bodily wastes, body contents, sick, deformed, dead or unhygienic people, some sexual behaviour, dirty environments, certain foods—especially if spoiled or unfamiliar—and certain animals [11,17,18]. Objects that have contacted any of the above can also become disgusting. Further, certain types of immoral acts are widely described as disgusting. Contact with disgust elicitors, real or imagined, is associated with (i) a characteristic facial expression that is recognizable across cultures [16,19], (ii) behaviour patterns that include withdrawal, distancing, stopping or dropping the object of disgust and shuddering [20,21], (iii) physiological changes including lowered blood pressure and galvanic skin response, recruitment of serotonin pathways, increased immune strength [22,23], and (iv) reports of negative affect including nausea.

Pathogen avoidance behaviour is universal across cultures, with all societies demonstrating individual and group-level hygiene behaviours. These include bodily, domestic and communal cleansing, avoidance of close contact or exchange of bodily fluids with others (with exceptions for mates and kin), and the avoidance of foods that are spoilt, contaminated or unfamiliar.

Explanations of disgust in the philosophical, anthropological, humanities and psychological literatures have been varied and inconsistent, reflecting this broad range of phenomena to be explained. The phenomenological philosopher Aurel Kolnai thought that disgust resulted from excess and surfeit: ‘A surplus of life, … an indifference to quality … a desire towards death’ [24, pp. 72–73]. Freud considered disgust a learned reaction formation that could be cultivated towards any activity through development [25]. The social anthropologist Mary Douglas argued that dirt and disgust are a product of culture, such that anomalous objects and events that do not fit the local cosmology have to be rejected, so as not to threaten social order [26]. In psychology, the dominant Rozin–Haidt school has disgust originating in the rejection of spoiled foods, but also serving to cope with the existential terror of being an animal and hence mortal [21,27]. A recent cultural study of disgust labels it ‘the Hydra’ since it seems too complex to explain [28].

An evolutionary perspective, however, provides a parsimonious explanation for the multiple elicitors and the behavioural tendencies of the disgust system. According to Darwinian thinking, disgust should be considered an adaptive system that drives the behavioural avoidance of infectious disease [11] Constant selection pressure from the ubiquitous presence of pathogenic parasites in animal and human ancestral environments [29] would have selected for those individuals with alleles disposing towards a ‘behavioural immune system’ preventing contact with, and incorporation of, pathogens [4]. Setting aside, for the present, the issue of moral disgust, it can be seen that all of disgust's basic elicitors (listed above) are implicated in the risk of transmission of infectious disease [11] and paired stimuli with, and without, disease risk show significant differences in disgust response [10,12]. This relationship between disgust elicitors and disease sources appears consistently across cultures and through the historical record [14,20,30]. Rats and other mammals display the characteristic gape expression after eating noxious food, and, as in humans, this reaction is dependent on the insular cortex [31] suggesting that disgust may be at least a pan-mammalian adaptation. Because behavioural immune systems are ubiquitous in animals and predate the evolution of modern humans, all humans should come equipped with a disgust system, rather than learning disgust as a product of culture (as Douglas, Freud and their followers have argued). It is also unlikely that disgust in humans originated in food distaste, because of the pan-vertebral need to avoid pathogens of all types, not just those using food as a vector of infection.

Brain imaging studies also show that there is a specific network associated with disgust. Viewing images of disgusting stimuli, or videos of people with disgusted expressions results in robust and recurring activation in specific brain areas—a neural network including the anterior insular cortex, basal ganglia, ventrolateral prefrontal cortex, anterior temporal cortex, medial prefrontal cortex and visual cortex [25,32,33]. Autobiographical recall of disgusting episodes activates the insular cortex and the basal ganglia [34], as does exposure to disgusting smells [33]. Two recent meta-analyses of functional neuroimaging studies of emotion found that activation of the basal ganglia was reliably associated with disgust [35].

## 3. The variability of disgust

Although disgust has an ancient and universal function, the disgust system reacts with different levels of activation to the same stimulus between individuals and over the lifetime of the same individual [27,36].

**Figure 1.**
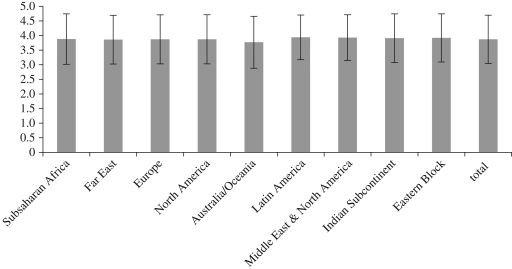
Mean and standard deviation of disgust scores for a sample of 38 845 individuals by region.

Figure 1 shows global variation in disgust sensitivity from a web survey with 38 845 participants. They were asked to rate how disgusting they found a series of disease-relevant images such as a sick person, a plate of what looked like bodily fluids and a crowded underground train, on a Likert scale of 0–5 (see [10] for details of methods). The results showed a high degree of variability between individuals (average standard deviation = 0.83). Differences in variability between continents were not statistically significant, varying only between 3.77 and 3.94 (s.d. = 0.05). Though a multivariate ANOVA model controlling for age, gender and occupational differences found cultural region to be a significant predictor of disgust sensitivity, this result was largely explained by the low average disgust sensitivity in the Australia/Oceania sample.

How do differences in disgust sensitivity arise? Evolutionary psychological approaches assume that psychological systems—including emotions—represent solutions to adaptive problems repeatedly encountered during evolutionary history. Plasticity in these systems should reflect the degree of variability in these problems: low levels of variability over the lifespan and between generations will favour the evolution of highly constrained systems. Such systems reliably develop in a broad range of environments with little phenotypic variation and have the benefit of efficiency and rapid and reliable development [37]. Greater environmental variation, on the other hand, should favour more plastic systems that use environmental—including social—information to adaptively shape behaviour according to the challenges of the local environment [38].

**Figure 2.**
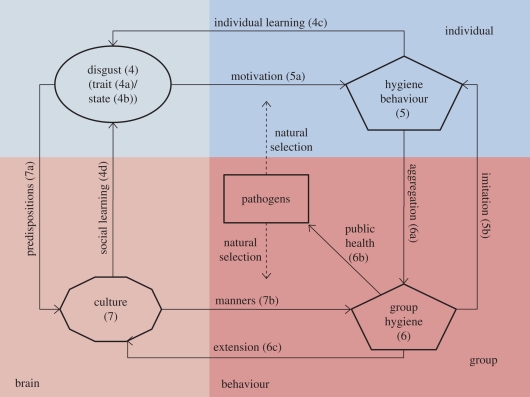
The disease avoidance adaptive system.

Pathogen pressure has led to the selection of a disgust/hygiene behaviour system in individuals that is both universal and plastic to local environmental variation. Figure 2 schematizes the factors that influence the human disease avoidance system. While disgust motivates hygiene behaviour, disgust sensitivity varies between individuals as a trait and within individuals by their state and also through individual learning. Disgust and hygiene in ultrasocial humans facing shared pressure from pathogens are not, however, simply a matter for individuals. The lower half of the diagram depicts group effects in the adaptive system. *Culture*—the socially acquired information shared by a particular group—affects the individual system through social learning and group hygiene behaviour through norms about *manners*. Group hygiene can be symbolically extended into cultural ideas about purity and pollution and can also affect public health by influencing the prevalence and virulence of pathogens in the environment. Group hygiene also influences the hygiene behaviour of individuals via imitation. The content of culture is, itself, a product of the individual brains that support it and so reflects the predispositions of those brains. Finally, pathogens exert selective pressure on the whole system.

All of the components of the system, whether in brains (shown on the left of the diagram) or in behaviour (shown on the right) are affected by environmental factors extraneous to the disease avoidance system, including seasonality, climate, ecosystem, habitat and the particular structure of host populations (not shown). In the following sections of this paper, we describe the elements of this adaptive system and how it works. (For ease of reference, numbers in the figure refer to the relevant sections of the paper.)

## 4. Disgust in brains: sources of diversity

Figure 2 shows pathogens exerting selection pressure on individual disgust and the hygiene behaviour it motivates. However, selection has not produced a constant level of disgust sensitivity. Variation can be accounted for by three kinds of phenomena: (i) innate trait differences; (ii) by plasticity, which allows for adaptation to current states; and (iii) the ability to learn from changes in the environment over the lifespan. These types of influence will be treated in turn.

### (a) Trait-based variation in disgust sensitivity

Individuals can consistently deviate from each other in behaviour because of stable trait-based differences. These arise from previous histories of adaptation in differing selective environments or trade-offs with other competing needs. A variety of traits are associated with variation in disgust sensitivity, including personality, gender and maternally inherited sensitivity.

The dominant theory of personality is the ‘Big Five’ approach, which suggests that stable, long-term patterns in behavioural proclivities can be summarized along five dimensions: extraversion, neuroticism, agreeableness, conscientiousness and openness [39,40]. Neuroticism is associated with a variety of deleterious traits such as an increased likelihood of experiencing negative emotions such as fear, sadness, anxiety and guilt [41]. High neuroticism is a strong predictor of psychiatric disorder in general, particularly depression [42], and is also associated with impaired physical health, presumably through chronic activation of stress mechanisms [43]. However, the reason for the persistence of such an apparently maladaptive personality trait may be that it helped to reduce the risk of predation and accidents in dangerous ancestral environments [44]. Given that there is covariance between neuroticism and disgust sensitivity scores [45] and that parasites were one of the biggest dangers in ancestral environments, it is probable that disgust sensitivity is, in fact, a component of the neuroticism trait. Indeed many animals display stable ‘personality’ traits [46,47]. For example, it has been shown that shy sunfish (*Lepomis gibbosus*) carry different types of parasite from bold sunfish, presumably because the differences in their behaviour mean that they are exposed to different levels of parasite risk [47].

Malfunctions of the disgust system can be seen as extreme trait variation. For example, some forms of obsessive compulsive disorder are thought to be related to excessive disgust sensitivity [48], while those with a genetic predisposition to Huntingdon's disease have been shown to have lower levels of disgust [49].

Men and women have recurrently experienced different costs of pathogen exposure. Disgust sensitivity varies consistently between males and females, with females consistently scoring substantially higher on measures of disgust sensitivity than males [10,27,50]. We hypothesized that this trait difference reflects women's differing history of responsibility for childcare [11]. Women, in effect, need to be disgusted enough for two people if they are to keep their dependent children free of disease.

### (b) State variation in disgust sensitivity

While individuals are born with varying disgust sensitivity traits (which may or may not be adaptive in current environments), there are also adaptive advantages to being able to modify one's disgust sensitivity according to one's current physiological state. There are times when one may be more vulnerable to pathogens; upregulating disgust sensitivity and hence concern for hygiene may thus be adaptive. Equally, there may be states in which it is advantageous to lower one's disgust sensitivity—when hungry or short of suitable mates, for example.

When physiological immunity is compromised, the probable costs of infection are greater and disgust sensitivity should increase. This interaction between physiological immunity and the behavioural immune system has been termed the ‘compensatory prophylaxis hypothesis’ by Fessler & Navarrete [51]. In one recent study, participants who reported more frequent infections had both higher disgust sensitivity and more ruminations about contamination and disease [52]. Women undergo adaptive immunosuppression after ovulation and during the first trimester of pregnancy in order for the maternal immune system to be able to tolerate the paternal genetic material in the blastocyst. Fessler *et al*. found stronger disgust responses to disease-relevant stimuli during the first trimester of pregnancy [53]. Increased nausea during early pregnancy is argued to fulfil a similar function, limiting exposure to toxins and pathogens during this vulnerable stage [54–56]. Because progesterone is the endocrine cue for a downregulation of immune response, Fleischman & Fessler [57] found that salivary progesterone correlated positively with disgust sensitivity and the frequency of rumination about disease and contagion in normally cycling women. Similarly, Conway *et al*. [58] found an increased sensitivity to social cues of disease (disgust expression plus averted eyes in another individual's face) when progesterone levels peak in normally cycling women. One further study found that opiate users make fewer errors in identifying disgust faces than non-, or ex-users [59]. Opiates have long been recognized as a powerful immunosuppressant [60].

State changes can also result simply from metabolic activity: after some time, the need to acquire additional resources increases, producing a state of hunger. Though hunger may lead to increased vulnerability to disease, it also signals that the benefit of caloric intake has increased in relation to the benefit of disease avoidance. This trade-off in disgust sensitivity is illustrated by a recent experiment by Hoefling *et al.* [61]. They found that, compared with satiated controls, participants who had not eaten food for 15 h showed significantly reduced facial expressions of disgust when presented with disgusting food stimuli. Interestingly, food deprivation did not influence facial expression of disgust in response to other disease-salient stimuli such as body wastes. The effects of long-term food deprivation have not, to our knowledge, been studied. Anecdotally, however, extreme hunger has been associated with cannibalism and other behaviours that would induce disgust in satiated individuals. It could, therefore, be predicted that chronic malnutrition would downregulate disgust for food but upregulate disgust for other potential sources of pathogens. It could also be expected that individuals who have been unable to secure suitable matings might downregulate their level of disgust towards a mating opportunity, thus trading the possibility of reproductive success for a higher risk of infection.

The perceived vulnerability to disease (PVD) scale has been used to glean self-report data from participants about both their perceived susceptibility and their aversion to germs [62]. The perceived infectability subscale, which asks about prior history of disease (e.g. ‘I have a history of susceptibility to infectious disease’) correlates with two different disgust sensitivity measures, indicating that the disgust system can be calibrated by previous states of illness (i.e. disease exposure).

### (c) Learning from hygiene behaviour

While disgust varies between individuals as a trait and within individuals according to their state, it is also important for the disgust system to be able to respond appropriately to specific features of the current environment. However, learning from pathogens is difficult; they are too small to be seen with the naked eye; they are costly to learn about through trial and error learning; they spread easily and imperceptibly; and their sources are highly diverse. Over evolutionary time, certain classes of stimuli have evolved warning ‘flags’—e.g. the smell of rotting flesh, the taste of faeces and the sight of deformity—thanks to the unique association between previous disease threat and a particular cue. Three learning mechanisms help animals to tune this innate system to current threats.

The first mechanism used by humans, and other omnivores, for responding to environmental variation in disease threat is known as the Garcia effect. Here, the experience of illness following ingestion of a food results in the ‘flagging’ of that food as aversive [63]. The Garcia mechanism is known to ‘misfire’: nausea induced by chemotherapy can result in long-term aversion to foods consumed before treatment [64]. More importantly from an evolutionary perspective, Garcia learning can only occur following illness. It is thus an efficient, but risky, way of establishing what is, and is not, a disease threat in the local environment.

Evaluative conditioning is a second learning process that results in the creation of stable disgust flags. Here, a powerful disgust reaction results in a previously neutral stimuli acquiring a disgust label (e.g. the sight of lumpy milk may fail to cause a disgust response until the milk is tasted and the disgust response to the bad taste then creates an association with the sight of lumpy milk). Labels like this are quickly acquired and slow to fade [65,66]; the process is succinctly described as ‘a sticky form of relational learning resistant to extinction’ in the title of a paper by Olatunji *et al.* [66]. Indeed, Baumeister [67] argues that this kind of learning is the primary function of the ‘hot’ affective component of the emotion.

A third disgust-specific learning mechanism is the ‘law of contagion’. When an object or stimulus flagged as disgusting touches another previously neutral object, this new object immediately acquires a disgust label, even if the contact is fleeting and no visible trace remains [20]. This learning system can thus track the spread of pathogenic contamination from surface to surface. Contagion-based labels are temporary and item specific: for example, a pen touched on a toilet seat will acquire a disgust label, but this will dissipate given time or washing, and other unrelated pens will not acquire this disgust flag [68].

### (d) Social learning from culture

Humans are ‘informavores’, seeking information about the best way to behave both from what others say and from what others do. Conspecifics provide a rich source of information about what is disgusting in the local environment. Social learning can make use of that information, avoiding the need for risky learning from direct contact with pathogen cues. This body of knowledge can then be readily passed on vertically from parent to child, and can also spread horizontally (within generations), given the human fascination with the disgusting [28,69].

An important way to acquire knowledge of what to avoid is to pay attention to specific social cues such as expressions of disgust in conspecifics. The fear literature suggests that experience of the expression of fear in a conspecific may be necessary for genetically ‘prepared’ stimuli (such as snakes) to become fear inducing [70]. A similar social triggering is probably required for some cues to become disgusting as well. The expression of disgust in another human elicits activation of the same specialized neural circuits that are activated by disgusting stimuli [33]; presumably this facilitates the acquisition of new disgust ‘flags’. A study by Bayliss *et al.* [71] examined this process by looking at how facial expressions influenced participants' evaluations of everyday objects. Objects paired with disgust expressions were rated more aversive than those paired with neutral or joyful faces. The ability of facial expressions of disgust to influence behaviour emerges early in life. Hertenstein *et al*. found that a 15 s verbal and visual display of disgust towards a novel object reduced infants' contact with a novel toy. In 14 month olds, the effects lasted at least 1 h [72].

Social learning may also be important in the reduction or removal of inappropriate disgust flags. In the domain of food choice, children are typically conservative in their preferences, with many foods classed as distasteful or disgusting. In an environment with adults and other individuals consuming food without disgust, negative evaluations fade and a more varied diet can develop [73]. This process can account for the wide cross-cultural differences in patterns of food preferences, as well as the ability to consume ‘off’-smelling foods such as durian and blue cheese, or unfamiliar animal foods such as witchety grubs or balut eggs. In the absence of appropriate social learning, benign or even healthy foodstuffs such as oily fish or green vegetables may continue to elicit disgust throughout life [73]. Analogous processes are common in non-human animals; Norway rats become willing to try a new food if they smell traces of the food on the coat of a conspecific [74]. A similar process may play a role in the reduction of stigma towards—and disgust of—people with physical deformity, for example. Experiments with infants show that positive attitudes by parents result in the dissipation of avoidance behaviours [75].

## 5. Hygiene behaviour

In figure 2 we show individual hygiene behaviour as a product of the disgust system in individual brains and also a product of the copying of the hygiene behaviour of others.

### (a) Disgust motivates hygiene behaviour

There is surprisingly little data on correlations between disgust sensitivity and hygiene behaviour. However, a recent study found that high disgust sensitivity predicted behaviour on only one of four behavioural avoidance tasks. The authors attributed this inconsistency to ceiling effects in their own experimental design. However, individual self reports of disgust during the task were a predictor of avoidance behaviour in each task [76]. Using more aversive behavioural tasks (touching urine, eating a cookie from the floor, etc.), Deacon & Olatunji [77] found that higher disgust sensitivity was associated with greater behavioural avoidance. Paul Rozin's many studies have demonstrated that participants who rated their subjective experience of disgust on paper as being particularly strong were more likely to refuse to touch a cockroach, less likely to eat unusual food items and less willing to touch body products like mucous [78].

### (b) Imitation: copy the successful, copy the frequent

Section 4*c* described how learning in the form of ‘do what I say’ can influence the disgust system. It is also possible for individuals to learn from the behaviour of others directly—learning in the form of ‘do what I do’. Humans use the behaviour of those around them as cues as to what are ‘fit’ strategies, using both the frequency of the behaviour [79] and the success of the model (e.g. wealth, high status and health) as guides [80].

We have some evidence that individuals do, in fact, imitate the hygiene behaviour of the group (though there are other effects here beyond blind copying). A study monitoring handwashing with soap in a motorway service station showed that it was more frequent if there were several people present in the toilet [81]. Similar effects have been found elsewhere [82].

Such blind copying can, of course be harmful. A review of handwashing studies in 11 countries found that handwashing with soap after defecation was rare (17% observed to do so on average over the studies). Respondents often claimed that handwashing with soap was not practiced because ‘it is not what we do around here’. The copying of the unhealthy, but frequent, variant was thus perpetuating risky behaviour in the group as a whole [83].

## 6. Group hygiene behaviour

So far we have looked at the effects of proximate factors on disgust sensitivity in brains and the hygienic behaviour of individual humans. Yet, humans are a social species; our behaviour reflects social as well as individual considerations. The lower half of figure 2 schematizes group-based effects on the disease avoidance system.

### (a) Aggregation

Clearly group hygiene behaviour is the sum total of the hygiene behaviours of individuals; this is schematized by the ‘aggregation’ line in figure 2.

### (b) Group-level hygiene behaviour and its effect on pathogens

Group hygiene behaviour is not, however, solely an amalgam of the behaviour of individuals. A social group acting together can engage in a wide range of cooperative strategies to reduce the transmission or the virulence of pathogens, or destroy their habitat. This is, indeed, the basic premise of public health. Individuals cooperate for the benefit of the group as a whole in, for example, sequestrating the sick, building and maintaining water and waste disposal infrastructure, adopting certain places and not others as defecation grounds, and in the provision of health services, such as vaccination, infectious disease treatment, the promotion of condoms and insecticide-treated bednets and the operation of food hygiene systems.

Public health is a common good: individuals who defect from paying the costs of contributing can still enjoy the benefits. Public health problems are, therefore, subject to free riding and the ‘tragedy of the commons’ [84]. The maintenance of cooperation with respect to public hygiene is notoriously difficult. In developing countries, community water supplies may work well for the first few years after they are constructed but then break down, for example. This is because it only takes a few individuals defecting from contributing to the on-going costs for cooperation to falter [85]. Various remedies have been tried. For example, in the 2009 H1N1 swine flu epidemic, healthcare personnel in the UK were implored to put the common good above their own fear of the individual effects of vaccination. Cooperative hygiene behaviour may need reinforcement in the form of sanctions or punishment—as we will discuss in §7*b*.

Group hygiene became especially important for humans living in close spatial proximity, as technological advances led to increasing urbanization [86]. While pathogens that spread directly from person to person may have tended to evolve towards lower virulence (it is to their own transmission advantage to keep their hosts alive and ambulatory), pathogens that spread through vectors such as mosquitoes or communal water supplies can evolve towards greater virulence. Ewald has presented evidence that one group hygiene behaviour, the chlorination of water supplies, has driven the cholera vibrion towards lower virulence because it now has to rely on interpersonal transmission rather than easy passage through faecally contaminated water [87].

### (c) The cultural extension of group hygiene

We have seen how group hygiene behaviour emerges from the hygiene behaviour of individuals, and in the following section we will see how norms serve to reinforce hygiene as a public good. But does the hygiene behaviour of groups also affect the content of culture? The large anthropological literature on the symbolic use of hygiene in the separations that cultures make between the pure and the polluted, the in-caste and out-caste, the sacred and the profane, suggests that it does. [26,88].

In figure 2 we have labelled as *extension*, the process by which hygiene behaviours with a biological function come to serve other cultural functions. So, for example, the act of washing took on symbolic significance in the lustrations and purification rituals of the early Semitic and ancient Greek religions, and later in Hinduism, Buddhism, Islam and Christianity. In ancient Egypt, priests were a group set apart by their immaculate purity; washing from head to foot twice every day and twice every night [89]. The Semitic religions employ rituals such as ‘Kippuru’: the washing off of a specially applied paste, to remove both material and moral pollution. Greek texts show how purification rituals could ‘cleanse’ the pollutions not just of childbirth, death and sex, but also of murder [90]. Muslims must bathe after defecation and prior to leaving the house, as well as symbolically, before contact with the divine in prayer.

The Laws of Manu, sacred texts of Hindu scripture (circa 200 BCE), prescribe the avoidance of the 12 impurities of the body: ‘Oily exudations, semen, blood, urine, faeces, the mucous of the nose, ear wax, phlegm, tears, the rheum of the eyes and sweat … ’ (Chapter V versus 135). While the biological basis for the avoidance of these substances is clear, the same text extends the argument to those whose work involves contact with polluting substances, making them a separate and polluted caste. These social distinctions may have their origins in biological avoidance strategies but then are made purely symbolic through invocation of the notion of ‘purity’. Thus, groups in power can label other groups ‘impure’. Untouchables can be argued to be infectious, hence disgusting and contagious. These associations are used to stigmatize the poor and the low caste. One's ability to stay physically clean—a measure of class—is extended to one's ability to remain morally pure.

The symbolic transformation from untouchable as ‘dirty’ to untouchable as ‘impure’ makes a group at one stroke not just biologically dangerous, but culturally dangerous. Rules of social distinction and separation become based on purely symbolic criteria, but are supported and sustained by the emotional resources of disgust and contagion [91]. With these rules of separation in place, one can then invoke moral indignation when they are violated, because such acts infect the social body with dangerous ideas of disease, sedition and corruption of the status quo, raising the possibility of the overturn of the social hierarchy.

Dietary laws can also be extended from biologically reasonable avoidances of particular animals as food to whole new classes of animals. Adherence to dietary rules then serves as symbols that demarcate members of one's own group from others [26]. The same logic of extension can be applied to relationships with outsiders: members of other groups can be both dirty (because they are likely to harbour unfamiliar diseases) and impure because they do not follow the same cultural prescriptions. Such xenophobia motivates solidarity within the group, which is helpful in inter-group conflicts, and so can be adaptive [92].

## 7. The effects of culture on disgust and hygiene

Culture consists of the pool of skills, attitudes, beliefs and values which have been socially learned by the set of individuals in a population or group [93]. Figure 2 shows culture affecting disgust via social learning (which we discussed in §4*b*). Culture also affects the hygiene behaviours that are adopted by groups (and hence, the behaviour of individuals) through the power of norms and manners. We also show group culture as, at least partially, a product of the biases of individual brains through predispositions to take on board certain ideas and not others. In modern societies, culture has also come to contain scientific ideas about pathogens and disease, and microscopic pathogens have finally become ‘visible’. We will discuss the consequences of these developments for the adaptive system at the end of §8.

### (a) Psychological predispositions affect culture

The content of culture is determined, at least to some degree, by learning biases: predispositions to pay attention to certain facts and not others [94]. Information about what is disgusting is salient to individuals because it can inform about how to avoid disease. Hence, individuals will pay attention to such information and will also preferentially pass it on to other individuals, if this can benefit their own fitness directly or indirectly. For example, the germ theory of disease may have come to be widely accepted globally because the idea of the spread of invisible disease agents accords well with the law of contagion and the predisposition it creates to be aversive to contamination [13]. Urban myths and etiquette rules that involve body wastes, animals or other disgust elicitors have greater ‘cultural fitness’ than non-emotional alternatives [95,96]. Although disgust is a negatively valenced emotion, and people generally avoid environments or people that elicit the emotion, humans also exhibit a clear fascination for the grotesque, the lewd and the filthy.

### (b) Manners

Groups have all sorts of cultural beliefs, attitudes and norms that can have major effects on behaviour. While beliefs and attitudes can be idiosyncratic, norms are commonly accepted rules for appropriate behaviour. Deviations from proper behaviour generally attract sanctions, such as shunning, fining or killing. Every culture has norms about hygiene behaviour, though cultures differ in which specific behaviours are regarded as hygienic, concerning, for example, table manners, willingness to engage in physical contact with others, bathing rituals, food taboos and caste divisions. Such rules determine the way cultures treat those who do ‘dirty jobs’ and the things they label as polluted. Culturally prescribed rules that limit contact with pathogenic substances, and thus support more instinctive avoidance behaviours, can be called ‘manners’. Manners such as keeping oneself groomed and hence ecto-parasite free, sneezing into a handkerchief, practicing safe sex or not defecating in public spaces serve to keep one's own infectious material away from others. While the emotional resources of disgust underpin these norms—as Nichols' analysis of sixteenth-century manuals of etiquette showed [96]—they can also serve to demarcate groups by caste, class or as ‘outsider’.

Hence individuals who do not maintain a level of individual hygiene behaviour sufficient to protect others have ‘bad manners’. They are commonly labelled as disgusting, can become subject to social sanctions (e.g. shunned), and thus lose the benefits that accrue from social life. Societies employ ‘hygiene police’ such as environmental health officers and border control officials to detect and punish those who cheat on pro-social hygiene rules—e.g. by selling contaminated food or importing potentially rabid dogs. Those who do not pay their water rates are fined or lose access to the benefits of clean water, and failure to address the need to sanction non-cooperators is one of the key reasons why village level water supply systems so often fail in developing countries [85].

## 8. Discussion: the disease avoidance system

In this paper, we have set out the components of the adaptive system that produces disease avoidance behaviour. We have seen that natural selection has produced a system that has taught individuals how to behave hygienically. Pathogens are, however, not just an issue for individuals, but for groups, and as a highly social animal, humans have group responses to diseases. Hence we have postulated a parallel system whereby group brains (culture) affect group behaviour as regards hygiene, and vice versa, and these can have important effects on the prevalence of pathogens. These group effects affect the brain and behavioural responses of individuals.

While we have set out evidence from many types of source about the individual links, is there any evidence of it acting as a complete system? Some intriguing new studies provide evidence that this is the case. It has been shown that societies faced with high pathogen pressure have higher average scores on personality traits such as extraversion and openness to experience [97], higher average scores on collectivism versus individualism scales [98] and higher numbers of religions [99]. These effects could not be explained by latitude, climate or socio-economic status. Further, in the first two studies, historical disease prevalence was a better predictor of these psychological variables than contemporary prevalence, suggesting that disease risk is a plausible cause, rather than a consequence, of the cultural differences observed. Taken together, these results suggest that societies that have faced high pathogen threats tend to become more inwardly focused, leading to less mixing and hence less contact with potential pathogen threats in the form of individuals from other groups.

Figure 2 suggests three ways in which the system may be operating to generate these effects. First of all, natural selection may have been in operation in the past history of the individuals in these groups, leading those under high pathogen stress to evolve higher levels of hygiene, driven by higher disgust sensitivity. This could have translated into lower willingness to engage with other cultures, and greater conservatism (which has been shown to be associated with conservative values [100]). However, when we compared the same disease prevalence data with our own disgust sensitivity scores from the dataset shown in figure 1, we found no significant correlations, which argues against genetic differences between groups owing to varying histories of pathogen prevalence.

The second way of explaining the observed relationships between pathogens and group ‘openness’ is that individuals are responding to the perception that there is a lot of disease about, i.e. sickness in others causing a risk, which drives individual learning, which modifies disgust responses, which shows up in lower openness scores and enhanced xenophobia. Navarrete & Fessler [101] observed that, not only does sensitivity to disgust predict more negative attitudes towards foreign peoples (xenophobia), but also predicts more positive attitudes towards one's own cultural in-group (ethnocentrism). They also showed that another disease-relevant individual difference variable—PVD—predicts both xenophobia and ethnocentrism. Further, xenophobic reactions to foreigners have been found to be stronger among people who feel personally vulnerable to germs and disease (as measured by the PVD scale) [102]. More particularly, results showed that higher levels of PVD predicted stronger anti-immigrant attitudes towards those from subjectively foreign locations. There was no such effect on attitudes towards culturally familiar immigrant populations. It thus seems probable that the intervening variable that can explain the observed relationship between pathogens and cultural characteristics is, in fact, disgust. A similar effect was observed in World of Warcraft (a web-based multi-player game). When a plague of ‘Corrupt blood’ began to kill up to half of the players, they began to avoid big cities [103].

A final possibility is that selection is operating on social groups. Those groups with the strongest collective anti-pathogen defences, such as strong norms about manners or avoiding strangers, outcompete those groups without. Using a model which carefully spells out the links in this adaptive system thus helps reasoning about the ways in which a complex set of results can be better understood.

The interrelated adaptive system we have described is not, of course, static. It exhibits positive feedback loops that can lead to interesting dynamics. For example, the proportion of hygiene cooperators in a population may fluctuate cyclically because as defectors from group-level hygiene activity become frequent, so will pathogens. When pathogen stress is high, it once again pays to cooperate, and the proportion of defectors should again decrease. The proportion of hygiene defectors may thus go up and down in response to fluctuations in the costs of cooperation. This dynamic has been formally modelled in the context of risky versus safe sexual behaviour: as the proportion of ‘safe’ strategists increases, the prevalence of sexually transmitted diseases (STDs) decreases. In this changed environment, ‘risky’ individuals may have some advantage. Oscillations of high disease/safe behaviour, low disease/risky behaviour thus occur [104].

There is also a positive feedback loop between culture and group hygiene by which cultural rules with real biological import (manners) can become subject to the symbolization principle we call ‘extension’. When a set of cultural rules becomes symbolic, they can extend beyond behaviour with direct relevance to biological pathogens. An obvious extension is from rules governing contact with biological parasites to rules governing contact with ‘social parasites’ (i.e. individuals that claim an unfair proportion of social resources). In this way, systems of manners can be extended to become systems of moral rules. Violators of rules for the apportionment of socially produced resources can then be labelled as disgusting, and sanctioned accordingly.

It is probable that there have also been major shifts in the way the system has operated since the dawn of *Homo sapiens*. Changes in environment, social structures and technology have led to changes in pathogen prevalence, as well as in individual and group hygiene. While the system operated ‘blindly’ for most of human history, Leewenhoek's invention of the microscope 300 years ago finally allowed us to ‘see’ pathogens. Pasteur & Koch popularized the idea that invisible germs caused disease, and the idea has now caught on globally, perhaps because the notion of an invading parasitic life form is so exquisitely disgusting [13]. The scientific method has also allowed us to determine the most effective and cost-effective methods of disease prevention, so as to inform health policy internationally [105]. We have thus moved from the instinctive practice of safe hygiene, to more educated approaches. However, the ancient emotion of disgust retains its power; a recent hygiene promotion campaign in Ghana successfully elicited disgust to increase rates of handwashing nationally [106]. We now plan to test whether promoting hygiene as good manners is an effective disease control strategy.

## 9. Conclusions

We have seen that disgust, hygiene behaviour and culture form an interlinked adaptive system, which has long served to reduce the dangers of disease. We have argued that disgust in the brain and the disease avoidance behaviour that it motivates is universal in humans (and in other animals) and is a product of the selection pressure of pathogens in the environment. However, disgust is also plastic, being able to retune according to signals from within the body and from the social and biological environment. Heightened disgust sensitivity leads to heightened disease avoidance behaviour (hygiene). However, as a social species, group hygiene behaviour is not just an aggregate of individual behaviour but the consequence of individuals using each other as models to be imitated, and as resources for cooperation in healthy behaviours. Changes in group hygiene behaviour, including cooperative public health activities, can reduce pathogen prevalence. The tendency to defect on responsibilities to the group can be countered by norms imposed by culture. The content of culture is itself biased by learning mechanisms based in the disgust system in individual brains.

We have shown that humans are equipped with a series of highly specialized learning mechanisms and biases. Learning about disease (and disgust) is not the product of domain-general systems; features of these learning mechanisms clearly reflect the nature of the disease avoidance problem they interact with. But the informational inputs to these systems differ, both between people and across groups, as a consequence of local disease problems and the learning histories of the individuals in one's social group. Individual differences are therefore inevitable. This variation, however, is frequently presented as evidence that evolutionary explanations are inappropriate (e.g. [107,108]). On the contrary, variable responses in the disgust system are indicative of a rich evolved psychology capable of absorbing and integrating information from diverse sources, and generating adaptive behaviour in a wide range of environments.

The adaptive system depicted in figure 2 highlights the intricate interrelations between the content of individual brains and group brains (culture) and individual and group behaviour. We have used it to explore one human need—the avoidance of disease. However, humans have multiple evolved needs including a need for food, to avoid predators, to rear young, to maintain social affiliations, etc. [109]. Our framework should prove useful in integrating perspectives from across disciplines for other similar adaptive systems.

Infectious disease still remains a leading cause of mortality worldwide [110] and promoting safe hygiene may be one, if not the most, cost-effective means of preventing disease [105]. Evolutionarily informed work that can explain the causes of disease avoidant behaviour may offer vital clues as to how best to change environments and cultures so as to favour changes in group and individual behaviour, and hence to prevent this annual toll of infectious disease. We hope that those who seek to promote disease avoidant behaviours will recognize the power of using the disgust system's natural affinity for producing adaptive responses to disease threats when developing future public health programmes.
